# Stroke survivors partner in research: a case example of collaborative processes

**DOI:** 10.1186/s40900-022-00386-2

**Published:** 2022-09-10

**Authors:** Alyson Kwok, Deacon Cheung, Maysyn Gordon, Evan Mudryk, Patricia J. Manns

**Affiliations:** 1 Faculty of Rehabilitation Medicine, University of Alberta, 8204 112th Street NW, Edmonton, AB T6G 2G4 Canada; 2 Glenrose Rehabilitation Hospital, 10230 111th Ave, Edmonton, AB T5G 0B7 Canada; 3 Healthcare Improvement Team, Research Building, Glenrose Rehabilitation Hospital, 10230 – 111thAve NW, Edmonton, AB T6G 0B7 Canada

**Keywords:** Patient partner, Patient-oriented research, Stroke, CVA, Rehabilitation

## Abstract

The Canadian Strategy for Patient-Oriented Research supports the inclusion of patients as partners throughout the research process. Purposeful and meaningful engagement of patient partners after stroke can present unique challenges due to the potential impacts on cognition, communication, or mobility. The purpose of this paper is to provide a case example of working together with three individuals who bring their post-stroke lived experience, including one person with aphasia, from study design through to dissemination. The designed and executed qualitative research was the purpose of this collaboration; this paper describes the collaborative process rather than the outcomes of the original research. The Strategy for Patient-Oriented Research Patient Engagement Framework was followed to engage the patient partners fully as part of the research team. Patient partners were involved at regularly scheduled team meetings and provided guidance on key aspects of project design and decision-making. The patient partners provided robust and important contributions to many aspects of the research, including shaping interview questions, assisting with thematic analysis, and contributing to the dissemination of research findings. Effective team dynamics were fostered by focusing on the value of the lived experience knowledge, using best-practice communication strategies, as well as taking time for relationship-building and story sharing. With appropriate support and guidance, the individuals who have experienced stroke were valuable contributing members of our research team.

## Plain Language summary

Whenever possible, including patients as partners in the research process can improve the quality of the research and the relevance of the results. Sometimes, there can be challenges when engaging people with lived experience in health research. For example, after a stroke, some patients can have trouble moving, thinking, or speaking. The purpose of this paper is to describe the processes used by our research team, comprised of two researchers and three patient partners with lived experience who had knowledge and insights into surviving a moderate to severe stroke. The research was a graduate student project exploring what patients think of physical therapy tests and measures used post-stroke. This article describes the process, rather than the outcomes of that research. We collaborated through virtual meetings, which were held every time decisions about the research had to be made or where feedback was required. The patient partners improved the interview question guide, helped to analyze the data, and helped to communicate the research findings. We found several strategies that helped us to be successful, including focusing on the value of the patient perspective and insights, taking time to build relationships, and allowing time to share stories. We also took special care to follow recommendations about communication to make sure that all the patient partners understood the project aim and activities that we were working on together. We feel this paper shows how patients who have had a stroke can collaborate as valued members of health research teams.

## Background

Patient engagement in health research benefits both patients and researchers, with proposed outcomes such as patient empowerment, improved understanding of the research area, and improved research effectiveness [1]. A recent review found that out of 119 articles on patient engagement published since 2010, only 9 included patients as members of the research team [2] and none specifically focused on the post-stroke experience. Researchers still struggle to differentiate between patients as participants and patient partners [3]. The term ‘patient partner’ is used to broadly describe anyone with lived experience with a health issue who is involved in a research project in a capacity other than as a research participant [4]. Patient partners are recommended as a part of a patient-oriented research strategy [4], but there remains little guidance around specifically how to engage patients as partners. Including patient partners as active members of the research team can be a daunting prospect, particularly when the nature of the patient’s experience, such as a stroke, may lead to unique engagement challenges. Although several research studies have explored participatory methods for people with sensory or intellectual impairments, most of this research is with people with learning disabilities [5]. In addition to challenges with movement or cognition, stroke survivors may also have trouble communicating, a condition called aphasia. Careful planning is required to ethically involve patients with aphasia as partners in research [6] and a failure to genuinely work to engage patients in the research process may lead to tokenistic involvement [7].

Our research successfully partnered researchers and patients with lived experience of a stroke to design and execute a qualitative case study. The purpose of this paper is to explicitly detail how we were able to purposefully engage and include patient partners with post-stroke activity and communication limitations in a research project.

### Preparing for patient-oriented research

The purpose of the collaboration was to guide the research process to explore patient and therapist perceptions of physical therapy outcome measures post-stroke, using a qualitative case study design [8]. The Canadian Institutes of Health Research (CIHR) Strategy for Patient-Oriented Research (SPOR) Patient Engagement Framework [4] served as our framework for patient partner collaboration. We were fortunate to have guidance from the Alberta Strategy for Patient-Oriented SUPPORT Unit (AbSPORU) representatives, who helped us to plan out specific approaches to patient partner inclusion, beyond the general principles provided in the SPOR framework. The team consisted of two researchers and three patient partners. The study was a graduate student project for the lead researcher (AK) and as such, the research question was determined before recruiting patient partners.

Since the patient partners were considered part of the research team, our local ethics board did not require them to be a part of our ethics approval (i.e., they were informing the project design process, not contributors of data, and therefore did not have to sign consent to participate in the project). The requirement for ethics approval may vary depending on the vulnerability of the population and the specific role they are playing in the research process [9]. Recruitment of the patient partners commenced while awaiting Research Ethics Board (REB) approval. The lead researchers created an informal job description to guide recruitment and enable potential partners to determine if they were interested in participating. We were advised by AbSPORU representatives to include at least three patient partners on our team; a minimum needed to support each other and provide balanced perspectives.

Necessary patient partner qualities and characteristics were discussed by the researchers before starting recruitment. Patient partners needed to have experience with inpatient and outpatient physical therapy after a stroke since that was the population we were studying. In addition to partners who were excited about participating in the research process, we also looked for a diversity of lived experience, background, and stroke outcomes, and specifically worked to include a patient partner with aphasia. Patient partners were actively recruited by phoning existing patient contacts who had participated in quality improvement or education projects with one of the researchers. We also circulated our request to local stroke physical therapists and asked for recommendations. Potential patients who were determined to be a good fit for the research team were contacted by phone initially by one researcher to explain the purpose of the study and what being a patient partner could include. This process follows a traditional patient partner recruitment model, with health system recruitment processes, as described by Vat and colleagues [10]. We followed up with an email briefly explaining the details and encouraged the potential partners to take a few days to consider the offer.

All three potential partners contacted agreed to collaborate as a part of the research team. Two partners had worked previously with one or both researchers and one had no prior existing relationship with the researchers. The partners included two men and one woman, aged 28 to 48. Their strokes occurred between 2 and 10 years prior to the study and each experienced extended inpatient stays after their stroke (between 6 and 9 months), with subsequent outpatient rehabilitation. One partner had experienced aphasia since their stroke, impacting both expressive and receptive communication. All three had hemiplegia and two walked with assistive devices in the community.

Terms of reference (“Appendix A”) were drafted and circulated before the first meeting, outlining expectations and roles. The patient partners were provided the opportunity to adjust the terms of reference prior to agreeing to the work. We asked the patient partners to be involved in decision-making, analysis, and dissemination, anticipating that the work would take approximately 20 h over at least a year. For compensation, we worked with the partners to determine what was fair and appropriate within our budget limitations, as per recommendations in the Strategy for Patient-Oriented Research Patient Engagement Framework [4]. The partners were offered an honorarium paid through money transfer or gift cards, depending on what was preferable to each partner. We also budgeted to reimburse any direct expenses that may have accrued during the project work, such as parking or childcare. However, no such costs were incurred during the study.

The process of including patient partners in the research process was primarily through regular team meetings. The planning, data collection, analysis and dissemination of this research took place from 2020 to 2021 and due to concerns about the spread of COVID-19, our team elected to hold all meetings virtually using Zoom. Meetings were arranged at least a week in advance, accommodating the patient partners’ schedules and preferences (one partner found it much easier to participate later in the day and our schedule was adjusted accordingly). All meetings lasted 60–90 min, which was manageable for each partner. The lead researcher facilitated the meetings, which were held at key points in the research process, where decision-making, adjustments, or analysis was required. Seven meetings were held in total. All meetings were recorded and transcribed to provide documentation of our collaborative process for reference.

To accommodate communication issues, we followed the suggestions laid out in, “Qualitative data collection: considerations for people with Aphasia” [11] and were able to correspond with the authors for further guidance. We used several suggestions from this article to support communication, such as breaking messages down into smaller pieces, speaking slowly, and using visual aids [11]. All meeting agendas were sent out at least 72 h in advance using plain language with keywords bolded. The lead researcher engaged in email conversations if any partners had questions before or after the meetings. The team also supported each other during conversations by allowing time for each other to respond and encouraging questions if further clarification or rephrasing was required.

Providing training and encouraging cross-communication are actions that support meaningful engagement [12]. We elected to incorporate training into the existing meeting structure for two purposes; this format ensured that the training was provided ‘just in time’ to facilitate application and meant that we were better able to stay within the bounds of our agreement in terms of hours worked. Presentations about the study purpose, terminology, and research design were split up over the first two meetings. Subsequently, new concepts and questions were addressed organically in plain language followed by a discussion with all team members. The researchers devoted significant preparation time to ensure that they were able to provide appropriate, informal teaching in response to questions that arose during the meetings, rather than schedule additional training sessions. For example, a discussion of the patient-therapist relationship led the researchers to introduce the concepts of engagement and compliance. To support relationship building and to facilitate understanding, we kept meeting agendas simple and allowed plenty of time for informal discussion and story sharing.

### The research process

The patient partners were brought onto the team early in the study design process. A draft of the study design and data collection plan including interview questions was presented to the partners, and they were given the opportunity to discuss and adjust the methods. The researchers listened carefully to partner suggestions to determine if a different methodology was required, rather than try to educate the patient partners on all the options available. While there were minimal changes to the overall design, the patient partners were actively engaged in finalizing the interview question guide. Each partner piloted the interview questions with one of the researchers. We met as a group afterward to discuss initial impressions and then incorporated the suggestions to improve patient understanding and to help elicit answers that would better answer the research question.

Data collection involved patient and therapist interviews, chart reviews, and observations of treatment sessions. At the time, facility entrance was only granted to employees and current patients, significantly limiting any opportunity for the patient partners to be involved in data collection. Instead, one researcher performed all the data collection and kept a reflexive journal throughout the process. The team met after the first two interviews to discuss challenges. The patient partners discussed the concepts of patient engagement and compliance which led to some question revision to better differentiate these concepts in the interview. The partners also reworded a question that was sparking defensiveness amongst early participants.

Thematic data analysis was used to analyze the data. We initially struggled with how best to include the patient partners in the process of analysis in a way that was meaningful without feeling overwhelming. As a group, we decided to focus primarily on the analysis of the patient interviews, with less time spent on other findings of the primary study such as the chart reviews, observation field notes, and therapist interviews. We received significant guidance from AbSPORU to help develop the process outlined below.

1. *Initial coding.* The two researchers independently reviewed the entire data set and developed initial codes. The two met to review early thoughts, codes and emerging themes. Using NVivo software®, quotes were grouped according to the early themes.
2. *Patient partner transcript initial review.* Prior to reviewing any codes or themes, the patient partners each reviewed two transcripts from patients whom the researchers had felt were expressing different views. We met as a team to discuss the patient partners’ initial thoughts. At this meeting, the lead researcher also provided an anonymized, verbal summary of the chart reviews and observations.
3. *Patient partner in-depth transcript review.* The patient partners then divided the remaining patient transcripts so that each transcript was reviewed by one patient partner. The researchers also selected three therapist interview transcripts representing different points of view. Each patient partner reviewed one of these therapist transcripts.
4. *Theming.* Once the patient partners had reviewed their transcripts, we met again for an in-depth discussion about the transcripts. The patient partners were presented with a document that had quotes grouped by the early themes developed, however, the quotes were anonymized, and preliminary theme labels were removed. The patient partners were encouraged to regroup any quotes that didn’t seem to fit and then were asked to determine a theme label for each grouping. This occurred by consensus during the meeting and through email correspondence afterward.

**Fig. 1:**
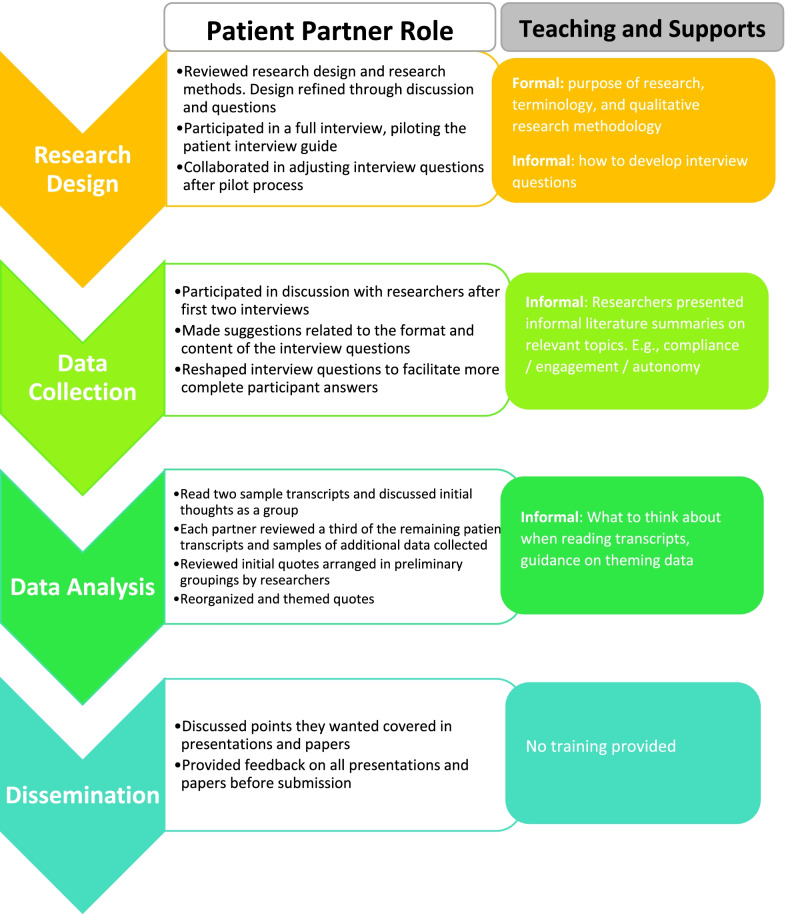
Patient partner role at each stage of the research process and supports received

Dissemination of the research findings occurred in three parts: a presentation to the research site, a manuscript of the original research findings, and this process paper on patient partner involvement. One of our final meetings occurred at the end of the research process to discuss how dissemination could occur , gauge interest and plan out the next steps. All partners were interested in being involved in all aspects of dissemination. We followed recommendations by Richards and colleagues regarding authorship [13]. The presentation and manuscripts were drafted by the lead researcher. The patient partners reviewed the initial drafts and any revisions before submission to provide feedback and agreement. For the site presentation, the patient partners actively participated in the presentation, answering questions and engaging in discussion with the therapists (Fig. 1).

The impact of patient-oriented research can be difficult to measure [14], however, we feel that our process had benefits for the patient partners, the researchers, and the research site. The impacts on our patient partners, researchers, and the research outcomes were assessed through informal discussion with stakeholders. The findings from our research project resonated strongly with our patient partners. They felt the resulting presentations and papers were representative of their experiences post-stroke and enjoyed having the opportunity to “give back” by participating in research that could benefit future patients. The researchers on the team appreciated the balance that the patient partners brought to discussions. The researchers were both physical therapists and felt a constant pull to attend to the therapist's perspective. The inclusion of patient partners led to an end-product that was patient-focused. At the research site, the patient-oriented research approach has elevated the importance of our findings and we have had the opportunity to begin additional, related quality-improvement and research projects.

### Reflections

Our research team worked to support one another, both through respectful communication and a flexible process. Meetings were structured around the patient partners and the researchers worked to actively remove barriers for the patient partners, such as adjusting meeting times to best suit the partners and assisting with understanding information circulated or presented in the meetings. The lead researcher responded quickly to email questions to support understanding or to capture an idea that might have suddenly occurred to one of the partners. Jargon was stripped or explained during all discussions and feedback was actively integrated into the next meeting. During each meeting, the team shared stories about their experiences, both patient partner experiences and the researchers’ stories of their clinical experiences. Initially, story sharing helped us to establish relationships with one another and function as a more cohensive team. Later in the research process, when we were analyzing participant interviews, story-sharing brought a different dimension to our research findings as we compared and contrasted participant experiences with our own. Bird and colleagues found similar facilitators to successful patient partnership, such as flexibility, using value-based action, empowering patient partners to share personal stories, using plain language, and creating strong relationships [15].

In this collaborative approach, we tried to work to everyone’s strengths. This meant finding new methods of analysis where the researchers performed some traditional coding and theming, while the patient partners reviewed and guided the work overall. While we felt this process was equitable [16], there was the potential for the balance of power to swing toward the researchers. To combat this tendency, the researchers iteratively interpreted and summarized the discussions, constantly checking in to make sure they were understanding correctly. However, as this was a graduate project, the patient partners supported leaving final decision-making with the lead researcher.

Our group was engaged and cohesive. The patient partners were animated yet highly respectful and compassionate. They were comfortable with disagreement and frequently used probing questions to better understand one another. They felt they could respectfully disagree because they all understood that everyone’s experience was different. Story sharing occurred organically and iteratively during each meeting. When one was sharing a particularly challenging story, the others provided encouragement through active listening and empathic comments. In general, we found story sharing to be a valuable tool. Even if the stories occasionally felt tangential, they helped the researchers to better understand the patient experience and helped us to connect with one another. To facilitate a gradual end to the patient partner-researcher relationship, involvement was tapered down, with communication occurring less frequently during the dissemination stages.

We experienced several challenges during our research. Due to the COVID-19 pandemic, we were unable to meet in person. The partners expressed that they would have liked to have some meetings in-person to further facilitate the relationship building. Fortunately, all the patient partners were very comfortable with technology. Virtual meetings had the benefit of eliminating travel time and the stress of dealing with parking. The patient partner with aphasia expressed that virtual meetings were more challenging than in-person communication, particularly if the audio quality was poor for one or more of the team members. Currently, live transcription is available for virtual meetings, but at the time, the software was difficult or expensive to obtain. In the future, we would recommend transcription or in-person meetings to support patients with aphasia.

Although we were able to roughly stay within the bounds of our work agreement (20 h), we felt our research would have benefitted from additional time with the patient partners. Additionally, we were only able to offer a small honorarium that did not properly compensate the partners for their time. We hope to be able to advocate for increased payment and additional patient partner time in the future by actively building patient partner compensation into grant applications. Our timelines stretched longer than originally anticipated, which can lead to compromised engagement during dissemination [17], but our full research team remained actively engaged.

For this project, the researchers had already determined the research question before the patient partners were brought on board. The patient partners indicated that if they had been involved from the beginning, they likely would have chosen a slightly different research question. They were most interested in the therapeutic relationship, rather than the therapeutic process. The patient partners also did not have the opportunity to be involved in any of the data collection. If we had chosen to use a focus group, rather than interviews, our patient partners were interested in participating as co-facilitators, which may have led to different peer-to-peer conversations.

Despite some of the challenges encountered, the patient partners had a positive experience. We encourage patients interested in research to consider collaborating as part of a research team. We would like future patient partners to understand that all contributions are helpful and suggest they try not to hold back or feel self-conscious. It’s helpful to discuss potential and unique barriers and for research teams to provide inclusive, inviting, and safe spaces to do so. Also, we hope potential patient partners recognize that partnering can have a personal benefit for patients as well as researchers.

### Patient partner reflection

Excitement, fear, and self doubt ran through me when I was first asked to be a part of the research team. I was excited in that I would be able to share more of my story and experience but at the same time I had reservations about how I would be able to contribute as I still felt a shadow of my former self. All the self doubt and questions of ‘Can I?’, ‘What do I know?’, and ‘What if?’ quickly vanished just by providing what I knew best of stroke and that was my lived experience. The best and easiest part was probably sharing with other survivors as that was our bond. Nothing needed to be said to understand what the others had been through. I would encourage others to get involved when given a chance as you can succeed by being open about your story and experience.

- Deacon

Helping Alyson during this process was empowering. Not only did I share my thoughts and ideas, but I also got to hear other, unique experiences. I knew I wanted to participate from the first contact we had. If by sharing my story, I can help others in their recovery or health care professionals, I will scream from the mountain top. I was honoured to be a part of this group and look forward to future collaborations.

-Maysyn

As the stroke happened with resulting aphasia, as a young person, I was determined to strive my very best abilities and not to be hindered but to be dynamic, thoughtful, and engaging. Thank you for giving me an opportunity to participate, Alyson and Trish, as well two non-aphasia stroke survivors around the group and the civil discussions. My aphasia is still with me, but as a writer, I embrace the challenges. I hope in the future to promote more of my motivational speaking engagements to increase awareness of stroke symptoms and resulting aphasia. I grapple with my own aphasia every single day and am trying to become more reaching, compelling and through provoking. I’m happy and proud of my own articles and drafts to become so far and beyond in the future. The honour is all mine and thank you for this opportunity, interesting and hopefully helpful.

-Evan

See Fig. 2.

**Fig. 2:**
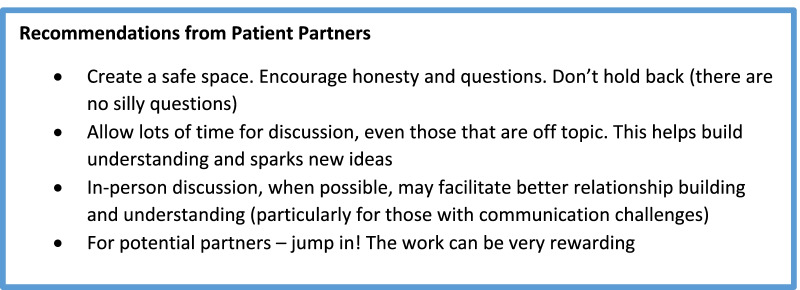
Recommendations from patient partners

## Conclusion

In the field of patient-oriented research, it can be difficult to authentically engage patient partners with a health condition that impacts their ability to collaborate in ways that are meaningful and equitable. Our research team successfully included three patient partners who had experienced a moderate to severe stroke within the last 10 years. Emphasizing relationship building and story sharing, while providing training and support were critical aspects of the partnership. The steps laid out may serve to help guide future teams as they endeavor to include patients as active and equal research team members.

## Data Availability

Data sharing is not applicable to this article as no datasets were generated or analysed for the comment article.

## Appendix A

### Maximizing patient engagement through collaborative outcome measures

#### Terms of reference

*Drafted September 2020*

#### About this document

- The terms of reference (abbreviated to TOR) will serve as a guideline for how our group will function
- All members can contribute and all need to agree before it is finalized

#### Goals

The goals of this group are to advise Alyson Kwok’s research for her master’s thesis. The group will provide the patient perspective to ensure that interview questions, focus group questions and conclusions reached during analysis are valid, sensitive and appropriate from a patient point of view.

#### Membership

- Alyson Kwok – Researcher (Chair)
- Trish Manns – Supervisor
- Evan Mudryk
- Deacon Cheung
- Maysyn Gordon

The role of patient partner will involve the following activities:

- Review and provide feedback on patient interview questions
- Review and provide feedback on therapist interview questions
- Review transcripts (with patient information removed), along with Alyson’s first interpretations and provide their opinions
- Review and provide feedback on focus group questions
- Optional: Members may choose to be present during the focus groups to listen to the discussion and help with co-facilitating (e.g. timekeeping and making sure everyone gets a chance to talk)
- Review transcripts, along with Alyson’s first interpretations and provide their opinions
- Optional: Participate in spreading the findings. This may include but is not limited to presenting findings to the staff at the local rehabilitation hospital and writing a manuscript for publication (members can choose what to participate in).

#### Group rules

- All members will openly and candidly share their ideas within the patient partner group
- All members will respect the opinions and time of others
- Any patient information shared will be kept confidential

#### Expectations

Participation in activities: Participate in the activities listed above, ideally by attending zoom meetings. If you are unable to attend a scheduled meeting, let Alyson know as soon as you can, and an alternative will be arranged.

Agenda: The meeting agenda will be sent via email at least 48 h in advance of the meeting.

Preparation: As we move further along in the process, you may need to read some information in advance, such as interview transcripts. This will be provided at least a week prior to the meeting.

Minutes: Minutes or notes will be kept from each meeting and can be distributed if members prefer to have a copy for their records.

Length of time: It is anticipated that the required activities will be completed sometime in January 2021. Dissemination (the spreading of the findings through presentations or papers, which is optional) may take several months afterwards.

Total time for participation: It is anticipated that participation in the required activities will take approximately 20 h from September 2020 to January 2021.

Withdrawing: Patient partners are free to withdraw participation at any time.

Decision Making: Final decision making will lie with Alyson Kwok and Trish Manns.

#### Reimbursement

If participation requires patient partners to send their own money (e.g. bus tickets or parking), the cost will be reimbursed.

#### Compensation for Time

Patient partners will be paid $200 for their participation. Members should communicate directly with Alyson if alternate arrangements should be made (e.g. if gift cards are preferred to an honorarium).

#### Research

Background:

- Physical therapists working with patients following a stroke often use standardized tests (outcome measures) to measure a patient’s improvement.
- Certain tests have been recommended with very little patient input.
- Conducting these tests can take a lot of valuable patient time.
- Outcome measures can help patients to understand how they are progressing toward their goal
- Connect Care may allow patients to see their own progress in the future

Purpose: The purpose of this research is to better understand how outcome measures are used in physical therapy after stroke and what improvements could be made to better help patients be more involved in their own care.

Settings:

Inpatient and outpatient stroke program at the local rehabilitation hospital.

Methods:

- Observations of patients and therapists participating in outcome measures and/or talking about goals
- Interviews with patients
- Interviews with therapists
- Focus group with patients

## Abbreviations

AbSPORU: Alberta SPOR Support Unit
CIHR: Canadian Institutes of Health Research
SPOR: Strategy for Patient-Oriented Research
